# Genetic and spatial characterization of the red fox (*Vulpes vulpes*) population in the area stretching between the Eastern and Dinaric Alps and its relationship with rabies and canine distemper dynamics

**DOI:** 10.1371/journal.pone.0213515

**Published:** 2019-03-12

**Authors:** Bianca Zecchin, Marco De Nardi, Pierre Nouvellet, Cristiano Vernesi, Massimiliano Babbucci, Barbara Crestanello, Zoltán Bagó, Tomislav Bedeković, Peter Hostnik, Adelaide Milani, Christl Ann Donnelly, Luca Bargelloni, Monica Lorenzetto, Carlo Citterio, Federica Obber, Paola De Benedictis, Giovanni Cattoli

**Affiliations:** 1 Department of Comparative Biomedical Sciences, Istituto Zooprofilattico Sperimentale delle Venezie (IZSVe), Legnaro, Italy; 2 Department of Infectious Disease Epidemiology, Imperial College London, London, United Kingdom; 3 Department of Biodiversity and Molecular Ecology, Research and Innovation Centre, Fondazione Edmund Mach (FEM), San Michele all'Adige, Italy; 4 Department of Comparative Biomedicine and Food Science (BCA), University of Padova, Legnaro, Italy; 5 Austrian Agency for Health and Food Safety (AGES), Institute for Veterinary Disease Control, Mödling, Austria; 6 Department of Virology, Croatian Veterinary Institute, Zagreb, Croatia; 7 Virology Unit, Veterinary Faculty, Institute of Microbiology and Parasitology, University of Ljubljana, Ljubljana, Slovenia; 8 National Institute for Health Research Health Protection Research Unit in Modelling Methodology, Imperial College London, London, United Kingdom; 9 Department of Statistics, University of Oxford, Oxford, United Kingdom; 10 Department of Veterinary Epidemiology, Istituto Zooprofilattico Sperimentale delle Venezie (IZSVe), Legnaro, Italy; 11 SCT2 Belluno, Istituto Zooprofilattico Sperimentale delle Venezie (IZSVe), Belluno, Italy; Faculty of Science, Ain Shams University (ASU), EGYPT

## Abstract

Information on the population dynamics of a reservoir species have been increasingly adopted to understand and eventually predict the dispersal patterns of infectious diseases throughout an area. Although potentially relevant, to date there are no studies which have investigated the genetic structure of the red fox population in relation to infectious disease dynamics. Therefore, we genetically and spatially characterised the red fox population in the area stretching between the Eastern and Dinaric Alps, which has been affected by both distemper and rabies at different time intervals. Red foxes collected from north-eastern Italy, Austria, Slovenia and Croatia between 2006–2012, were studied using a set of 21 microsatellite markers. We confirmed a weak genetic differentiation within the fox population using Bayesian clustering analyses, and we were able to differentiate the fox population into geographically segregated groups. Our finding might be due to the presence of geographical barriers that have likely influenced the distribution of the fox population, limiting in turn gene flow and spread of infectious diseases. Focusing on the Italian red fox population, we observed interesting variations in the prevalence of both diseases among distinct fox clusters, with the previously identified Italy 1 and Italy 2 rabies as well as distemper viruses preferentially affecting different sub-groups identified in the study. Knowledge of the regional-scale population structure can improve understanding of the epidemiology and spread of diseases. Our study paves the way for an integrated approach for disease control coupling pathogen, host and environmental data to inform targeted control programs in the future.

## Introduction

The red fox, *Vulpes vulpes* (order Carnivora, family Canidae) is a globally widespread and non-migratory species [1], which shows remarkable adaptability to a variety of habitats and food sources. It is native to Europe, Asia and North America. More than 40 red fox sub-species have been recognized and 2 of them are present in Italy: *V*. *v*. *crucigera* (in the Italian peninsula and Sicily) and *V*. *v*. *ichnusae* (in Sardinia) [2]. The red fox is usually monogamous and the home range of each family group is relatively stable. The size of home ranges varies from 40 to 700 ha in urban and suburban areas, reaching 1500 ha in forests [3]. Fox populations are not static, with seasonal patterns of dispersal. The number of dispersers depends on population density, home range and level of human activity and control. Dispersal distances are also extremely variable, ranging from 0 to more than 300 km [4], with a mean of about 40 km estimated in Sweden [3], with increased dispersal distances potentially increasing opportunities for infectious diseases to spread. Furthermore, the fox colonisation of urban and semi-urban areas may represent a risk to public and animal health for disease spill-over occurrence.

The red fox is currently considered the principal reservoir of rabies virus (RABV, genus Lyssavirus, family Rhabdoviridae) in mainland Europe, although the raccoon dog (*Nyctereutes procyonoides*), also sporadically present in north-eastern Italy, might act as rabies transmitter in eastern and central Europe [5]. Over the last decade, the incidence of wildlife rabies in Europe has been considerably reduced thanks to the eradication programmes promoted by the European Commission [6]. Nevertheless, the disease has been notified in countries previously considered free from sylvatic rabies (namely Italy, Montenegro, Greece, Macedonia and Kosovo) and it remains endemic in Eastern Europe, which highlights the need to uphold transboundary surveillance systems and the importance of supporting joint elimination programmes. In Europe, between 2006 and 2017, the total number of rabies reported cases in wild animals decreased from 6058 to 580 [7].

In October 2008 fox rabies emerged in northern Italy [8] more than a decade after the country had been recognized as terrestrial rabies-free; the newly achieved status prompted the implementation of a multiannual control programme that was implemented in coordination with the neighbouring countries (Austria and Slovenia). While Austria maintained its achieved free status [9], Slovenia, like Italy, experienced a new infection wave, with its peak in 2008–2010 [10]. Joint oral vaccination programmes of foxes led to the control of the epidemic first in Italy and then in Slovenia [7] and protected the Austrian territories from the disease. Similarly in Croatia, due to the implementation of the oral rabies vaccination (ORV) campaign in 2011, the number of rabies cases decreased consistently with no cases detected since 2015 [11, 12].

Apart from rabies, the red fox is well known to be infected with the Canine Distemper virus (CDV, genus Morbillivirus, family Paramyxoviridae), a pathogen of great relevance to wildlife conservation. CDV infects a wide variety of mammalian species [13, 14] but it predominantly spreads through the species belonging to the Canidae family, such as the red fox. Although distemper surveillance in Europe is poorly documented, CDV is widely circulating in Central Europe [15–19] persisting in Austria, Slovenia and Croatia (Table A in S1 File). An epidemic of distemper in wildlife spread throughout northern Italy in 2006–2010 [20]. After this first epidemic, CDV re-emerged in far eastern territories from 2011, with cases currently still being detected. From late 2008, both diseases were circulating in the same Italian regions. Rabies and distemper cases were confirmed in red foxes with a percentage of 84% and 82% respectively, while other wild species appeared to have played a minor role in the epidemics [20, 21].

In the last decade, genetic data have been widely used to investigate the structure, gene flow and mixing among individuals of a given animal species [22–25], which has greatly improved our knowledge of the connection between wildlife and landscape features [26]. Understanding the population dynamics of a reservoir species may help to predict the dispersal patterns of infectious diseases, which in turn informs the design and implementation of disease control programs [27–30]. Concerning the red fox in Europe, population genetic studies indicate a high gene flow and weak or total absence of a genetic structure of the red fox at European level [31, 32]. By contrast, a remarkable degree of differentiation among red foxes in the Mediterranean area, as well as within specific European areas was described in other studies (e.g. Britain-Ireland-Holland *versus* the Iberian Peninsula; or northern Europe *versus* eastern Europe *versus* single European countries) [33–34–35]. Similarly, independent studies on population genetics of the red fox in limited geographical areas found a low degree of differentiation in Switzerland, Poland and Croatia [36–38]. Clustering of the red foxes was detected in both Israel and United Kingdom [39–40].

In this study, we investigated the genetic structure of the red fox population in a wide geographical area stretching between the Eastern and Dinaric Alps. We also evaluated the association between the diseases (canine distemper or rabies) in north-eastern Italy and different fox subgroups and we discussed the most likely reasons for our findings. To our knowledge, this is the first study, which has used microsatellite analyses to investigate the red fox population genetic structure in relation to a combination of two different diseases, rabies and distemper, thus representing an innovative approach to improve the surveillance and control of infectious diseases on a regional scale.

## Materials and methods

### Study area and sampling scheme

The study area includes the northern regions of Italy and the territories of Austria, Slovenia and Croatia, covering an area of 200,596 km^2^.

Between 2006 and 2011 fox samples (*V*. *v*. *crucigera)* were collected for the diagnosis of rabies and CDV and a phylogenetic characterisation of CDV circulating in north-eastern Italy has already been documented [20]. Since late 2008, samples have been collected in northern Italy as part of both the passive national surveillance strategy for rabies and the assessment of the efficacy of the oral rabies vaccine. Collection of samples was carried out according to the European Food Safety Authority (EFSA) guidelines outlining the monitoring and reporting scheme for rabies [41], the OIE Manual of Diagnostic Tests and Vaccines for Terrestrial Animals 2018 [42] and the technical EU report [43]. All of the samples were tested both for CDV and rabies viruses; they were classified with respect to their CDV and rabies infection status and particular attention was given to the selection of samples from animals living close to the Austrian and Slovenian borders. Samples from Austria, Slovenia and Croatia were collected between 2010 and 2012 by the Austrian Agency for Health and Food Safety (AGES), Institute for Veterinary Disease Control, Mödling, Austria, the Institute of Microbiology and Parasitology in Ljubljana, Slovenia, and the Croatian Veterinary Institute in Zagreb, Croatia. The main goal of the purposive sampling was to focus on particular characteristics of interest of the population. A non-random selection of these samples allowed the selection of a uniform sub-sample across the years and the affected regions. The final sample size (n = 627) accounted for the laboratory capacity and for the condition of samples at collection. We then selected 379 Italian fox samples from the original panel of available specimens collected between 2006 and 2011, 98 Austrian fox samples collected between 2011–2012, 86 Slovenian fox samples collected between 2010–2011 and 64 Croatian fox samples collected in 2012 (Table A in S1 File).

### DNA extraction and microsatellite genotyping

Due to the genetic similarity between dogs and foxes, a set of canine microsatellite markers was adapted for the *Vulpes vulpes* genome [44–46]. Total genomic DNA was purified from frozen (-80°C) brain tissue by a DNeasy Blood & Tissue Kit (QIAGEN) according to the manufacturer’s instructions and stored at -20°C. Some of the primers for dog homologues, chosen among the ones previously published, were redesigned (hereby indicated with a *) as per Moore et al. (2010) [47] and Sacks et al. (2011) [48]: AHT-121 [49]; *RF-CPH2, *RF-CPH3, *RF-CPH11, *CPH18 [44]; AHT-137, *C01-424, C04-140, *C08-618, *FH2001, *FH2010, *FH2088, *FH2328, *FH2848, *RF-REN105L03, *RF-*REN162C04, *RF-REN169O18 [50]; *RF-CXX-279 [51]; *RF-INU055 [52]; RF-CXX468, *RF-CXX402 [53]. Forward primers were fluorescently labelled (6-FAM, VIC, NED, PET; MWG-Operon). As per the Sacks protocol [48], polymerase chain reaction (PCR) amplification was carried out in a DNA Thermo Cycler 9700 (Applied Biosystems) in 25 μl of the reaction mixture containing 25 ng of template DNA; similar or higher amounts of DNA were used by Wandeler et al. 2003 [36], Moore et al. 2010 [47] and Mullins et al. 2014 [37]. We conducted PCR in six multiplex groups using the Qiagen multiplex kit (QIAGEN), according to the following PCR profile: initial denaturation step at 95°C for 15 min followed by 35 cycles of denaturation for 30 sec, annealing at 58°C for 90 sec, extension at 72°C for 1 min; a final extension step at 72°C for 10 min. Each PCR amplification included a positive sample, required as a reference for the subsequent microsatellite analysis. Contamination was excluded by means of blank extractions and PCR-negative controls. PCR products were diluted (1:200 multiplex 3 and 6; 1:300 multiplex 1 and 5; 1:400 multiplex 2 and 4) to avoid any problems of peak artifacts due to an excess of DNA and subsequently analyzed on an ABI PRISM 3130*xl* automatic sequencer, with Genescan 500 LIZ (Applied Biosystems) as an internal size standard. The molecular size of microsatellite alleles was evaluated by using GeneMapper4.0 (Applied Biosystems). Genotyping was checked by re-amplification and analysis of 10% of the samples for each multiplex PCR. The error rates between replicates for all loci were low (1–2%). To minimize scoring errors two operators independently read and edited the program output.

### Genetic and spatial characterization of the fox population structure

We tested the reliability of 21 microsatellite loci. We evaluated the presence of null alleles and of genotyping errors due to large allele drop-out or stuttering with Micro-checker, adopting a 95% confidence interval for the Monte Carlo simulations [54]. We also estimated the number of alleles per locus, the values of observed (Ho) and expected (He) heterozygosity and the F index (F = (He-Ho)/He) with GenAlEx 6.5 [55]. The deviation from Hardy-Weinberg equilibrium (HWE), for each locus and globally, was tested using a Chi-square test, in GenAlEx 6.5 [55].

To improve our understanding of the spatial and genetic structure of the fox population we performed a spatial autocorrelation analysis and we assessed the presence of isolation by distance through the Mantel test, considering the genetic and spatial distances at individual level. Both tests were implemented in GenAlex version 6.5 [55]. We also investigated the isolation by distance at cluster level and the methodology is explained in the subsequent sections. The spatial autocorrelation analysis was used to assess the correlation of genetic and geographic distance at multiple distance classes [56]. The test generated an autocorrelation coefficient (r) and its 95% confidence interval (95% CI), which provides a measure of the pairwise genetic similarity of individuals whose geographic separation falls within a specified distance class. The significance of the analysis was determined by the heterogeneity test, based on 999 random permutations [57] and whose significance was set at p < 0.01 as per Peakall’s (2012) recommendation [55]. The heterogeneity test challenged the null hypothesis of no spatial genetic structure.

The population structure was investigated using a Bayesian clustering analysis implemented in Structure 2.3.3 [58], which allowed us to infer the number of clusters and visualize the memberships of the individuals. HWE and linkage equilibrium were assumed; we used an admixture model with correlated allele frequencies and without prior information on the population. Six independent runs for each K (number of inferred clusters, ranging from 1 to 10) were carried out using a Markov Chain Monte Carlo (MCMC) with 1,000,000 iterations (burn-in of 250,000 iterations). The number of clusters was determined according to Structure harvester, implementing the Evanno method [59, 60]. Clumpp 1.1.2 [61] was used to align the 6 repeated runs of the best K value obtained by Structure. To assess the presence of isolation by distance at a cluster level, we used the Isolation by Distance Web Service [62]. Taking into consideration the clusters identified by Structure, we tested the matrix correlation between genetic [Fst/(1-Fst)] and geographic distances, with 1000 randomizations to assess significance.

In order to measure the genetic differences between the identified clusters, we calculated the pairwise Fst values (probability based on 999 permutations) and performed an Analysis of Molecular Variance (AMOVA, using GenAlEx 6.5). This analysis tested the null hypothesis that the clusters are part of a single randomly mating genetic population. We performed these analyses for the individuals with cluster membership Q ≥ 0.7 [63]. Using Geneland 4.0.2. [64], on an R 2.15.0 platform [65], we spatially characterized the clusters and inferred their genetic structure. Similarly to Structure, Geneland assumes that the putative groups are at HWE with linkage equilibrium between loci. It identifies the population structure as systematic deviation in allele frequency from HWE and linkage equilibrium predictions, thus delineating their spatial organization. In the model, the spatial organization of the populations is assumed to follow the coloured Poisson-Voronoi tessellation [66, 67] and the allele frequencies are drawn from the Dirichlet distributions and are assumed to be either independent (uncorrelated spatial model) or non-independent (correlated spatial model) [67, 68]. As recommended by Guillot et al. [67, 69], we performed the Bayesian inference using MCMC simulations applied first to the uncorrelated and subsequently to the correlated spatial model. We inferred the optimal number of clusters (K) between 1 and 10 based on highest average posterior probability based on 200,000 MCMC iterations (100 thinning, 200 burn-in). Five runs were performed for each model to test the reproducibility of results. Finally, the geographical parameters were set to account for the study area boundaries.

In order to visualize the genetic clusters (as per Structure results) and to explore the presence of landscape features (e.g. natural or artificial barriers) which may have influenced the spatial characterization and movements of the individuals belonging to different clusters, we generated and assessed integrated maps showing genetic clusters as well as rivers and highways using ArcGIS 9.2 (ESRI, Redlands, CA, USA).

The number of different alleles and of effective alleles, the observed heterozygosity (Ho), the expected heterozygosity (He) and the F index ((He-Ho)/He) for different groups and clusters were obtained by using GenAlEx 6.5.

#### Ethical statement

This study was performed on samples collected and submitted to the national reference laboratories for rabies within the framework of the national rabies surveillance plans. The investigation did not involve endangered species, neither the intentional killing of animals. All samples originated from wild red foxes found dead or legally hunted. In Europe, such procedures do not require any specific ethical approval as hunting plans are organised by national authorities in the framework of disease control programs, according to the EU guidelines and to the national vaccination control plans approved by the EU [70]. The sampling procedures were performed in compliance with the country’s own legislation and the recommendations of international institutions [70]. According to the national legislations regulating animal experimentation, no ethical approval or permit was required for collecting and processing the type of samples examined for this study.

## Results

The 21 selected microsatellites had an average of 12.6 alleles per locus (range: 4–23; SE: 1.1). The observed (Ho) and expected (He) averaged heterozygosity per locus were relatively high (with means of 0.75 and 0.79, respectively) and the mean F index was 0.05 (range: 0.01–0.11). Considering the whole population, six loci significantly departed from HWE (FH2010, C01-424, FH2328, CPH18, RF-CXX402, FH2088); in the presence of multiple clusters, loci that show a deviation could be the most informative, as they reflect the very population structure.

**Fig 1:**
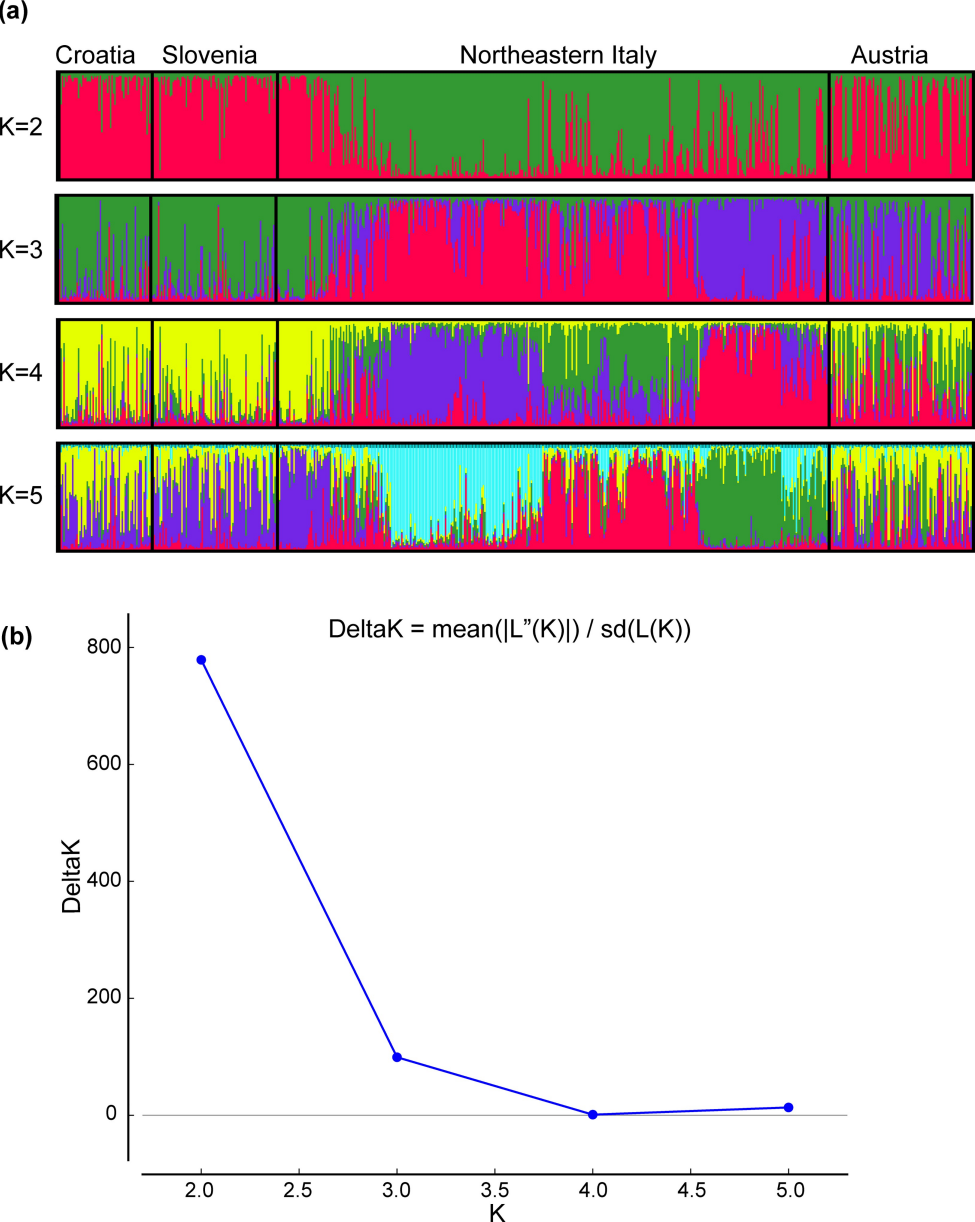
Structure results for the whole dataset. (a) Structure graphs generated by Distruct 1.1 [89]. Cluster membership according to the analyses of 21 markers in the dataset (627 individuals), for K = 2–5. The individuals are presented along the x-axes. The thin vertical line represents the posterior probability (Q from 0 to 1) for each individual to belong to a different cluster. Different colors represent the membership to a specific inferred cluster. Groups A and B are colored in red and green, respectively (K2). A line with no clear assignment to either cluster (e.g. a line with no dominant colour) is considered as an admixed (or migrant) individual. (b) Structure analysis estimated K = 2 as the most likely number of clusters.

**Fig 2:**
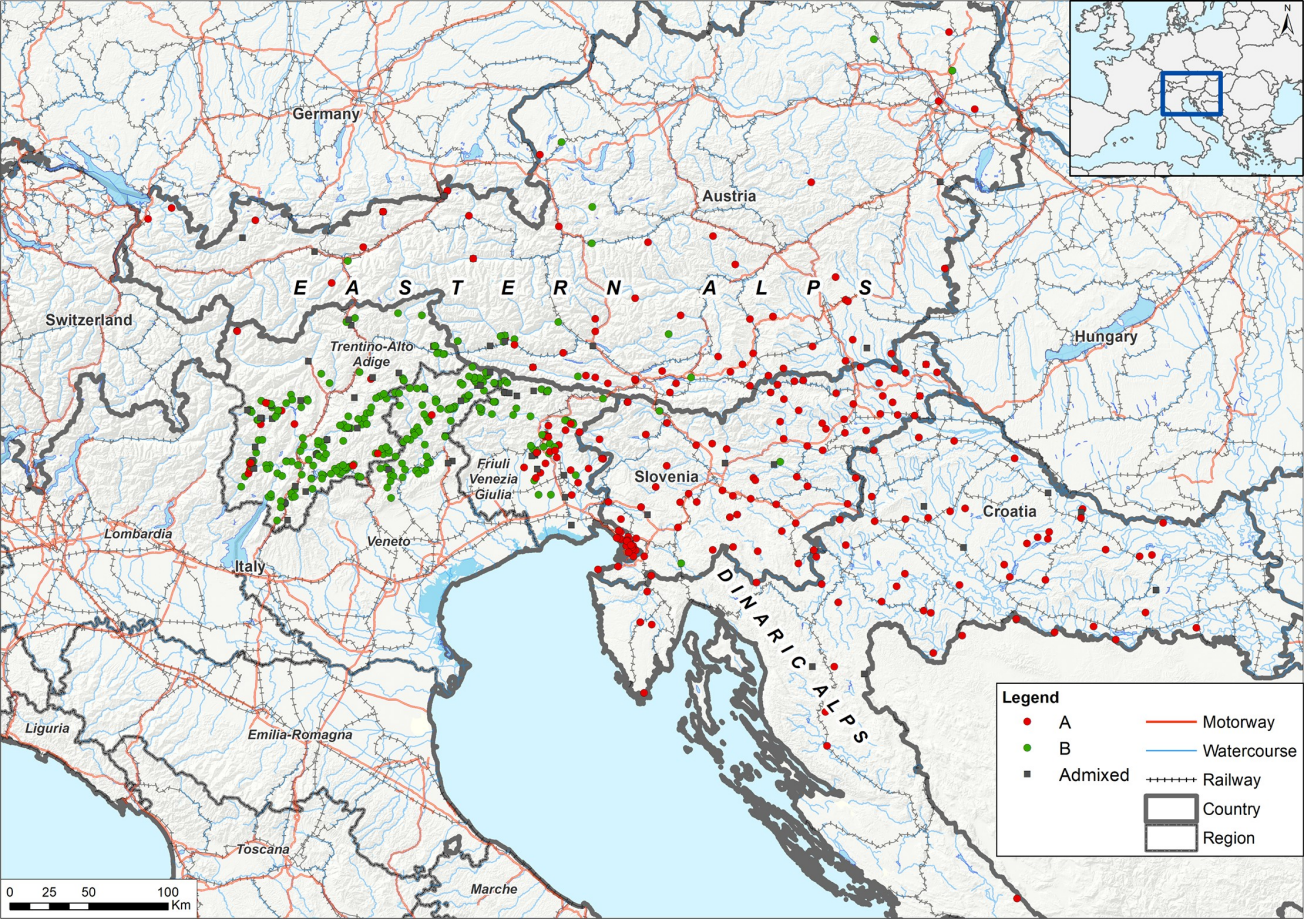
**Distribution of the genetic groups (A-B) in the study area.** Individuals assigned to group A and B are identified with red or green dots, respectively. In the map, samples with an assignment probability greater than or equal to 0.7 (Q ≥ 0.7) are shown with dots; admixed individuals are shown with grey squares. Mountains (Eastern Alps and Dinaric Alps) are shown in the map. Motorways, railways and water courses are shown in red, grey, and light blue respectively.

The number (K) of possible clusters was estimated in Structure assuming no prior knowledge about structuring in populations of individuals. The maximum likelihood estimate for the number of clusters was K = 2 (Fig 1). The assignment of genotypes resulted in two groups, namely A and B (Fig 2), consisting of 296 and 331 individuals, respectively. The majority of Austrian, Slovenian and Croatian individuals (71%, 96.5% and 98%, respectively) and of far eastern Italy (Trieste and Udine provinces) belonged to group A. Group B mainly consisted of Italian foxes (79%) while very few Croatian and Slovenian individuals (<4%) and 29% of the Austrian samples belonged to this group. The mean number of different alleles, effective alleles, observed heterozygosity, expected heterozygosity and F index for groups A and B are shown in Table D in S1 File. Considering the two groups (A and B) two loci departed from HWE (FH2010, CPH18); We therefore repeated the analyses with and without these loci and obtained the same results in Structure.

The result for K = 3, although less probable according to Structure results, was also biologically meaningful. In this case, the three groups of individuals, namely, A, B and C (S1 Fig), were all present in North-eastern Italy, occupying the Far Eastern, the Central and the Western territories, respectively (Fig 1 and S1 Fig). Of note, clustering of individuals from Croatia, Slovenia and Far East Italy was confirmed, while results from population structure of Austrian individuals was more difficult to interpret, with most individuals plotted as admixed and only partially clustering within group A and C (Fig 1 and S1 Fig).

**Fig 3:**
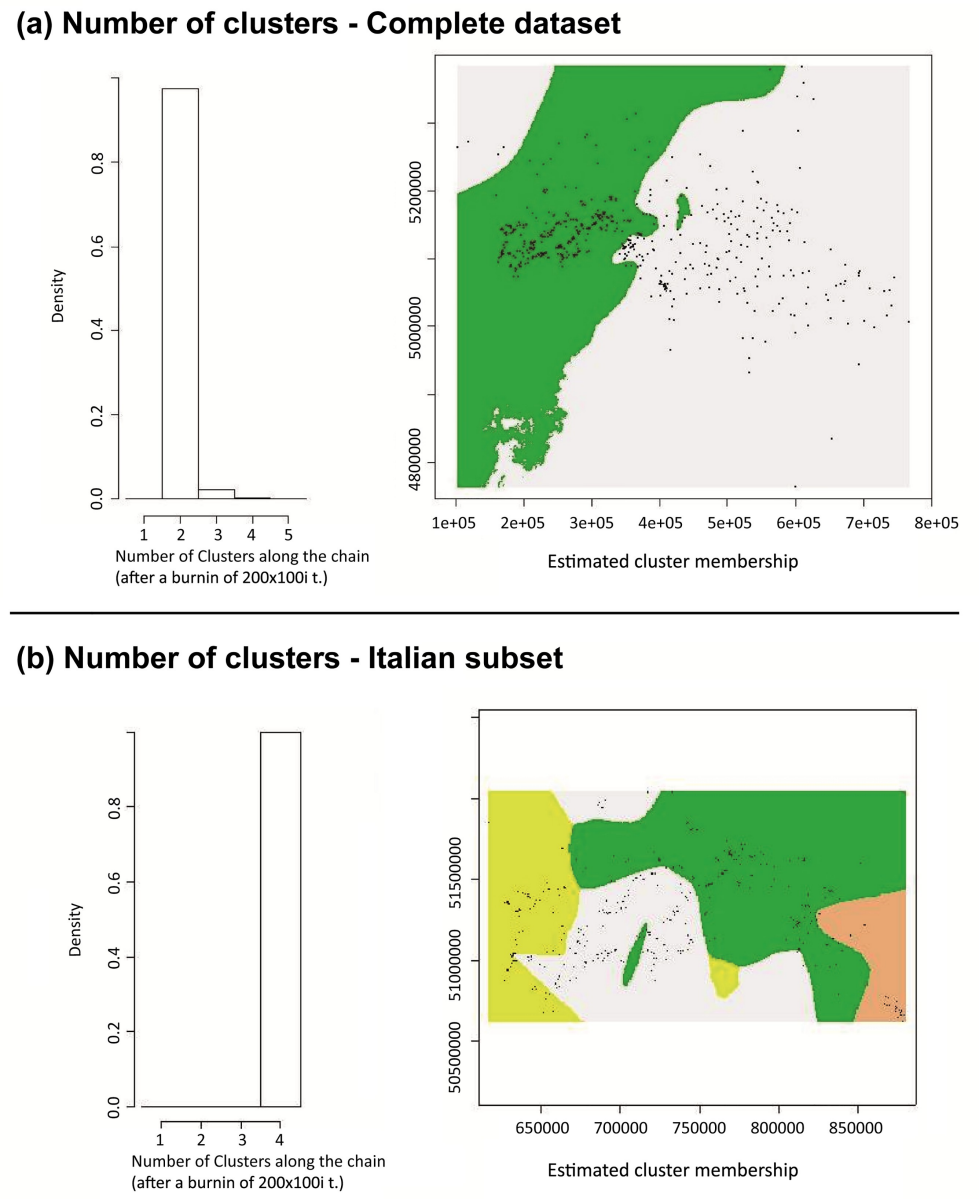
Spatial model results (Geneland). **a)** Geneland results on the complete dataset. **b)** Geneland results on the Italian subset. Starting from the left, the first graphs show the maximum estimate of K (clusters) simulated from the posterior distribution. The maps on the right show the spatial characterization of the identified (K) clusters (mode of the posterior probability of belonging to each cluster). Samples are shown as black dots.

The pairwise Fst value ((Ht-Hs)/Ht, Ht = total expected heterozygosity, Hs = average within population heterozygosity) showed a very low but statistically significant (p<0.001) differentiation among group A and group B (Fst = 0.009). The results of AMOVA showed that the highest percentage of variation was found within the study area (94%), while differentiation among clusters was very low but significant (2%, p<0.001). Geneland analysis supported the hypothesis of a weak population structure. The uncorrelated spatial model identified 2 clusters (K = 2) (Fig 3, section a). In the correlated spatial model, the highest posterior estimate of K was not identifiable. Assigning the number of clusters to “2” as per Structure (Fig 1), the spatial characterization resembled the outcome of the uncorrelated model.

We then focused on the Italian subset as the three disease statuses (either as rabies infected, distemper infected or non-infected) were all present for individuals collected from this area only. No animal showed co-infection of distemper and rabies.

**Fig 4:**
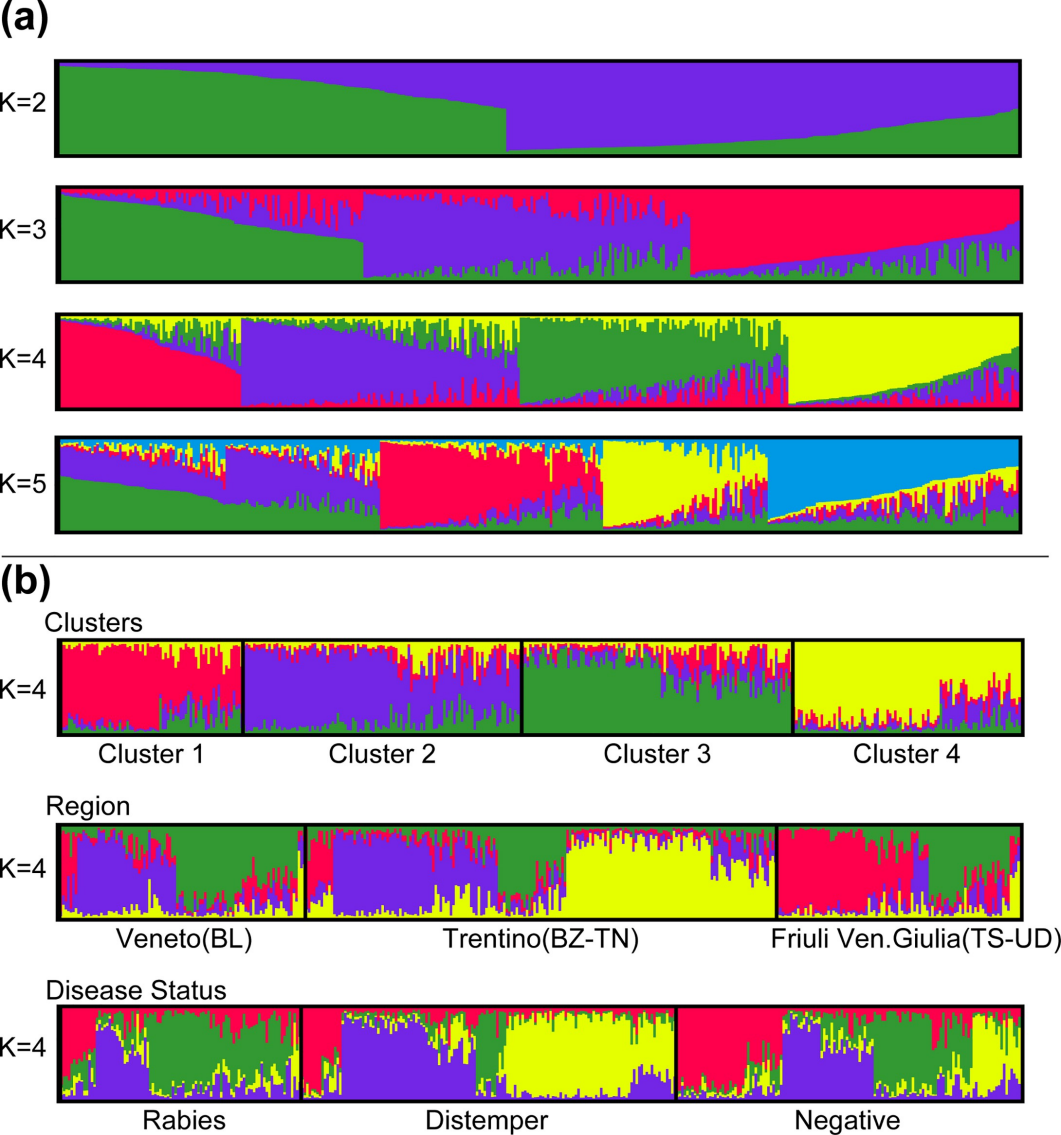
Structure results for the Italian dataset. Structure graphs generated by Distruct 1.1, [89]. (a) Cluster membership according to the analyses of 21 markers (379 individuals), for K = 2–5. The individuals are presented along the x-axes. The thin vertical line represents the posterior probability (Q from 0 to 1) for each individual to belong to a different cluster. Each inferred cluster is represented by a different color. Individuals are sorted by Q. (b) **Top.** Individual allocation to each genetic cluster. Different colours represent the membership to a specific cluster (Cluster 1: red, Cluster 2: blue, Cluster 3: green, Cluster 4: yellow). A line with no clear assignment to either cluster (e.g. a line characterized by different colours with no dominant colour) is considered an admixed (or migrant) individual. **In the middle.** Assignment of genetic clusters according to different regions and provinces. **Lower.** Assignment of genetic clusters according to disease status.

**Fig 5:**
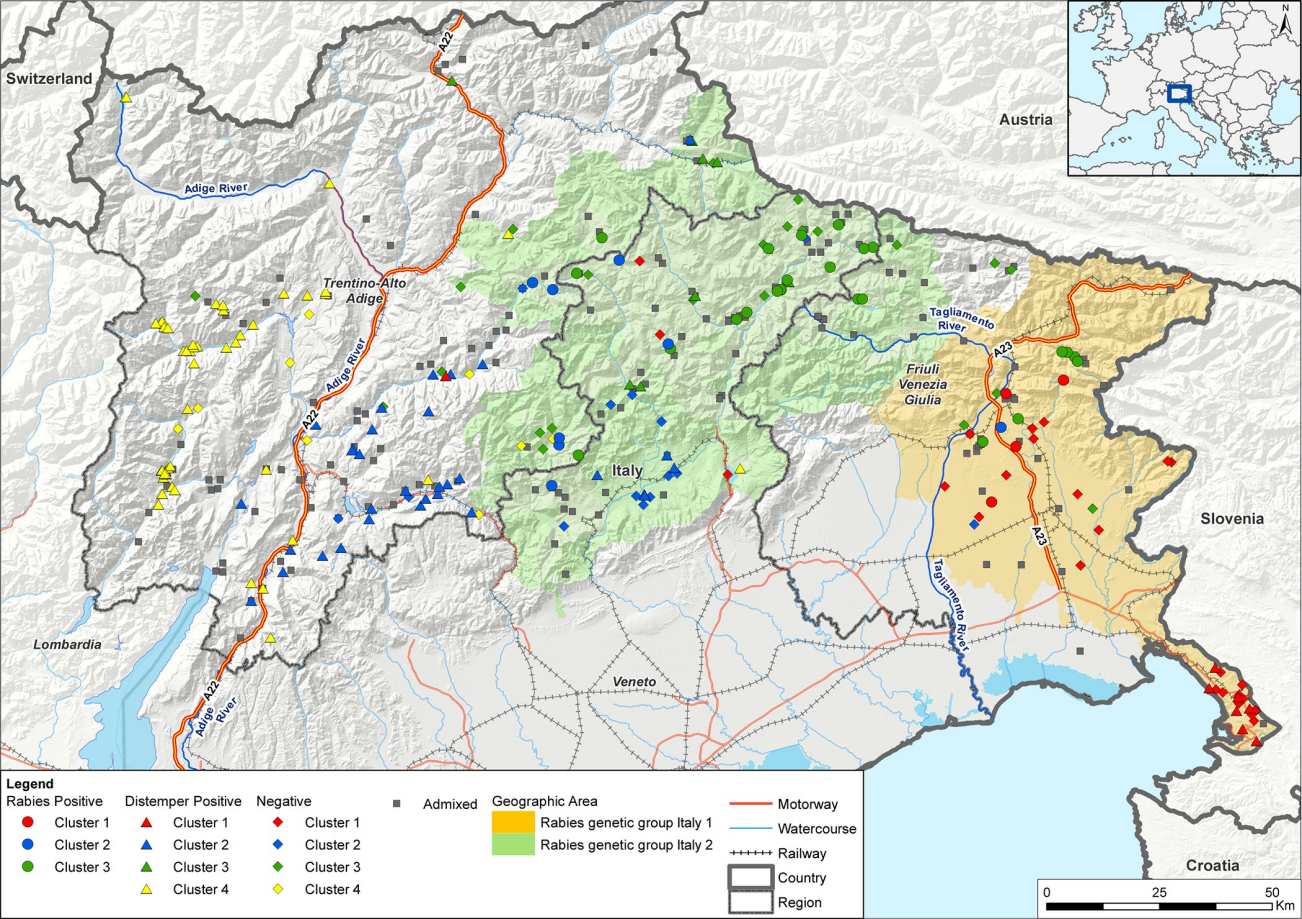
Distribution of the genetic clusters (1–4) in north-eastern Italy. Individuals assigned to clusters 1, 2, 3, 4 are respectively identified in red, blue, green, and yellow. Rabies positive samples are identified by circles, distemper positive samples by triangles and negative samples by rhombus shapes. Admixed individuals are presented as grey squares. The light orange area shows the territory occupied by individuals positive for rabies genetic variant Italy 1; the light green area shows the territory occupied by individuals positive for rabies genetic variant Italy 2. Motorways, railways and water courses are shown in red, grey, and light blue respectively.

The Structure analysis estimated that the Italian fox population was most likely divided into 4 clusters (Fig 4, Fig 5, S2 Fig). According to the maximum likelihood allocation 70, 112, 106 and 91 individuals were assigned to 4 groups, which were arbitrarily called cluster 1, 2, 3 and 4, respectively (Fig 4, Fig 5). Assignments of genotypes were non-randomly distributed in space. Among geographical areas, clusters 2 and 3 dominated the Veneto region; clusters 2 and 4 were mostly present in the Trentino Alto Adige region whereas clusters 1 and 3 dominated Friuli Venezia-Giulia (Fig 4, section b). The pairwise Fst values calculated on the Italian samples showed that the differentiation among clusters was very low but statistically significant (p<0.001). Considering the individuals with membership probability Q ≥ 0.7, cluster 2 appeared to be marginally closer to clusters 3 and 4 (Table 1). We observed slightly higher Fst values when comparing cluster 1 against all the others (Table 1). These observations were supported also when adopting Q ≥ 0.8 membership probability (Table B in S1 File), which showed the highest differentiation between cluster 1 and all the others and the lowest differentiation between cluster 2 and cluster 3/4 (Table B in S1 File).

**Table 1:** Genetic differentiation between pairs of red fox clusters.

	Cluster1	Cluster2	Cluster3	Cluster4
Cluster1		0.001	0.001	0.001
Cluster2	0.039		0.001	0.001
Cluster3	0.037	0.032		0.001
Cluster4	0.035	0.031	0.037	

Pairwise values of Fst in the lower triangle of the matrix and p-values in the upper triangle, among four clusters composed by individuals with membership probability Q ≥ 0.7 (GenALEx).

The mean number of different alleles, effective alleles, observed heterozygosity, expected heterozygosity and F index for clusters 1, 2, 3 and 4 are shown in Table E in S1 File. Considering the four Italian clusters, three loci departed from HWE (FH2010 for clusters 1 and 3; C01-424 for clusters 2 and 3; CPH18 for clusters 3 and 4); We repeated the analyses in Structure with and without these loci and obtained the same sub-structuring.

Considering the subgrouping of the Italian samples in clusters 1, 2, 3 and 4 towards the two main groups A and B we found that 89.2% of cluster 1 was included in group A, 100% of clusters 2 and 96% of cluster 3 were included in group B while cluster 4 belonged to group A (30%) and B (70%) as per the reported percentage values.

We then stratified by disease status (Fig 4, lower section b) and found that rabies had affected clusters 1, 2, 3 while CDV had affected mainly clusters 2 and 4. Secondly, we stratified by rabies variant only fox individuals infected by rabies. Briefly, two viral variants were circulating in Italian foxes during the recent 2008–2011 outbreak, according to the classification from Fusaro et al. (2013) [71], namely Italy 1 and Italy 2. Of note, each viral group, which had first been introduced into Italy through Friuli Venezia Giulia westwards, occupied different geographic areas during the epidemic indicating further distinct migration patterns. As expected, we confirmed that rabies infected individuals, belonging to cluster 1, were affected by Italy 1 viruses [71]; on the contrary, Italy 2 viruses [71] segregated into clusters 3 and, to a lesser extent, cluster 2, although firstly introduced into cluster 1.

The spatial autocorrelation analysis showed a positive correlation up to 80 km (r = 0.004–0.051, *p* = 0.001, S3 Fig). The genetic distance was not significantly correlated with geographic distance.

We found no evidence for isolation by distance among red foxes in the entire area (Mantel test, R^2^ = 8E-06, *p* = 0.39, S4 Fig)

## Discussion

Using both routine surveillance and research activities focussed on distemper and rabies, we obtained a large sample size on which to undertake our analyses of the genetic and spatial structure of the fox population in the area stretching between the Eastern and Dinaric Alps encompassing northern Italy, Austria, Slovenia and Croatia. We successfully adapted canine genetic markers to the genome of European *Vulpes vulpes*, and used the adapted markers to look for structuring within our dataset (S1 Dataset).

Based on the ecological knowledge of species habitat (red fox as a non-migrant but highly mobile species) we overall expected a weak genetic differentiation of the red fox population. Furthermore, due to the absence of co-infections of the two pathogens observed in northern Italy [21], we hypothesised that the landscape architecture of this area could have influenced the distribution of rabies and distemper in different clusters. Based on previous evidence of at least two rabies variants with different geographical spread in northern Italy [71], we expected a correlation between the introduction of the pathogens and the population genetic structure. Similarly, we assumed that the population structure of the different regions target of the study might have influenced the spread of the infectious diseases by limiting the movement and interaction of individuals.

Findings from our analysis of the complete dataset (n = 627) were consistent with observations of previous works, which revealed the presence of a single fox population characterized by a faint structure [31–34, 72]. Besides this overall finding, subgroups analysis indicated very modest genetic differences between groups of animals, which appear partially separated in the survey area by natural and artificial barriers (Fig 5).

Based on the results obtained, we were able to identify two genetic groups, that we arbitrarily named as A and B. The group further east (group A) extends mainly across Slovenia, Croatia, eastern Austria and the far eastern Italian territories located on the border with Slovenia. Group B includes few Austrian foxes but mainly consists of Italian samples and covers the remaining territories of the study area (provinces of Trento, Belluno and Bolzano) (Fig 2). Such a population classification was confirmed by the analysis of the Italian subset alone, in which we were able to confirm the existence of a far eastern group (cluster 1) and a central-western group (clusters 2, 3 and 4) consisting of Italian individuals belonging to group B. In summary, we identified Friuli Venezia Giulia region as a border area in which circulating individuals are genetically more similar to those from Slovenia and Croatia (cluster 1/group A) than to those of the remaining areas of north-eastern Italy (cluster 2-4/group B). As a support to our results, a phylogenetic analysis on the cytochrome b and D-loop by Statham et al. 2014 [35] identified a clear differentiation between Italian red foxes and the fox population circulating in the Balkans and Eastern Europe.

Based on the fox behaviour, a highly vagile species [40] that can disperse long distances [73], we expected very low Fst values between groups A and B. Our study confirmed this hypothesis as the pairwise Fst value was significant (p < 0.001) but very low and indicated a weak differentiation among foxes belonging to different groups; this is in line with previous findings from the literature. As the red fox population structure observed in Alaska, a much larger study area of more than 1,700,000 km^2^, is characterised by eight clusters comprising two main regions and much admixture with Fst values ranging from 0.006 to 0.059 [74]. Of note, limited levels of population differentiation were found when population genetics were studied at a national scale, such as in Switzerland [36], Poland [37], Croatia [38] and Portugal [72]. Our results can be also compared to those found in a study performed in Israel, which identified four genetic clusters with a genetic diversity expressed by a mean Fst value of 0.043 [39]. A recent investigation on the red fox in the Great Britain showed the existence of genetic diversity (Fst values ranging from 0.004 to 0.181) and a population genetic structure consisting of different genetic clusters [40]. Differences in observed patterns are likely reflecting landscape heterogeneity, land use, the existence of physical barriers, the interaction with other species and the distribution of resources [75–78].

The two groups identified in the area stretching between the Eastern and Dinaric Alps are geographically separated by natural and artificial barriers. Group B seems to be confined in Italy by the Western and Eastern Rhaetian Alps of the state of Tyrol in Austria and the Carnic Alps and the Karawanks of the Carinthian lands. In the Friuli Venezia Giulia region, the Tagliamento river and the fenced highway running along the river (A23) (eastern barrier) seem to separate group B from group A. In support to our results, particular geographic features and barriers, such as narrow land passes (70 km wide) combined with mountain ranges higher than 1000 m, have been previously held responsible for limiting fox migration and inhibiting gene flow, consequently leading to a pronounced genetic structure in Croatia [38]. The occurrence in the South of Austria of individuals belonging to group B might be due to the presence of corridors and crossing points that facilitate the passage through the Alps. A gateway could be identified in the valley of the Drava River, which on the other hand could represent a barrier to the north, and in the region of Carinthia, which is a basin within the Alps and could facilitate the movements of foxes between Austria and Slovenia.

Focusing on the Italian sub-set, the four genetic groups (clusters 1 to 4) identified were distributed in three zones of the target area, namely eastern (cluster 1), corresponding to group A, and central/western zones (clusters 2–4), corresponding to group B. Of note, two clusters were located in the central zone occupying the northern and southern parts of the study area, between the eastern (Tagliamento river and highway A23) and western barriers (Adige river and highway A22). As for findings obtained from the entire dataset, although the genetic differentiation of the four clusters is low based on the pairwise Fst values obtained, such a spatial characterization seemed to be relatively stable, with each identified cluster consistently present in the same zones across the whole study period (2006–2011). Assignment probabilities for each cluster showed evidence of admixture and revealed a limited gene flow between the eastern and central areas and between the central and western zones, although a high gene flow characterized the whole population (Fig 4, Fig 5). This result highlights the adaptability of foxes to different habitats and their potential for dispersal [1, 4, 31]. The impact of the artificial and natural effective barriers has already been investigated in relation to the distribution of the European roe deer (*Capreolus capreolus*) in northern Italy [75] and chamois (genus *Rupicapra*) [76]. Our results suggested that the presence of geographical barriers (e.g. rivers, motorways and mountain ranges) likely impedes dispersal and somehow limits gene flow in foxes. A similar finding and interpretation is also described in Japan where one out six cluster of red foxes located in the Hokkaido Island was mostly differentiated from the others due to the topology of the peninsula [79]. As for Japanese foxes [79], our population structure on the small geographical scale of north-eastern Italy is likely to reflect the presence of artificial and environmental barriers able to reduce but not to prevent movement patterns. For example, in the eastern zone of our study area, the eastern barrier seemed to have played an important role in reducing the gene flow between cluster 1 and cluster 3 (Fig 5). In the western zone, the western barrier seemed to have represented a strong limitation to dispersal between clusters 2 and 4 (Fig 5).

In relation to the distribution and spread of rabies and distemper in foxes, our findings might help explain and possibly forecast the two-pathogen disease dynamics in this area. Stratifying the individual classification according to disease status (Fig 4, lower section b), we noted an association between structure and disease, with cluster 1, 3 and, to a lesser extent, cluster 2 more affected by rabies, while cluster 4 and 2 by distemper. The different geographic distribution of Italy 1 and Italy 2 rabies variants affecting cluster 1 and cluster 3, respectively, is a clear evidence of limitation in individual contacts. Similarly, the unlikely movement of group B individuals from Italy to Austria through the Alps could have been partly responsible for the maintenance of a rabies free-status in Austria despite the occurrence of the disease in North-Eastern Italy [8], although Austrian oral vaccination preventive campaigns might have played an important role as well. However, although reducing the gene flow, natural and artificial barriers have proven not to be as efficient as preventing viral spread. This has been highlighted by the presence of admixed individuals, not clearly assigned to any cluster, reflecting the nature of highly mobile organisms such as the red foxes. Of interest, Italy 2 rabies variant, primary introduced in Far East territories, was able to cross the eastern barriers and in late 2009 occupy the central area affecting mainly cluster 3 individuals. Of note, the 2009 oral rabies vaccination of the Far East Italian strip between the eastern barrier (A23 and the Tagliamento river) and the Slovenian border was not able to prevent the westward spread of rabies during the last epidemic [71]. Similarly, distemper was first notified in spring 2006 in the province of Bolzano and mainly spread in the western cluster individuals, but was able to cross the western barrier as well [20]. The virus responsible for the 2006–2010 distemper epidemic fell into a monophyletic genetic group belonging to the Western European clade, which is extensively circulating in Central and Western Europe [20]. Based on the epidemiological and phylogenetic analyses, the epidemic might have arisen from a unique viral introduction through movement of infectious individuals [20]. Unfortunately, further information about the genetic characteristics of circulating distemper viruses were neither timely nor retrospectively provided from the area object of our study and from Switzerland, thus preventing any further speculation on the origin of such an epidemic. Of interest, in recent years (2011–2018) in northern Italy, distemper spread mimicked the previous rabies epidemic, although with a much slower transmission rate [21]. The infection was indeed able to cross the eastern barrier, thus spreading westwards again (manuscript in preparation).

Findings from our study are in line with previous evidence in the north-eastern United States demonstrating the dampening effect of rivers on rabies spread [80, 81]. Interestingly, other works suggested that differences in permeability of rivers to raccoon gene flow was associated with different rabies incidence in Ontario, Canada [82]. Of note and similar to our findings about the segregation of Italy 1 and 2 rabies virus variants, the population structure of the artic fox (*Vulpes lagopus*) in Alaska mirrors the distribution of the three known rabies virus variants [74].

The distribution and spread of rabies and distemper proved to be quite different and varied according to the areas of the territory object of our study. For instance, rabies did not expand across the Adige River while distemper reached the most western boundaries. Although the transmission potential of the two diseases in the north-eastern Italian setting have been estimated as equal (as for R_0_ 1.26), rabies has a shorter generation time so that its spread is much faster than that of distemper [21]. The absence of evidence of rabies infection in cluster 4 (far west) was likely due to the vaccination implemented, more than to other environmental factors. Thus, the geographical segregation of cluster 4 might have only served in part to protect it from the recent rabies epidemic. A number of factors, not directly investigated in this work, might have had a role in influencing the distribution of these diseases in the fox population object of our research, including the impact of rabies vaccination. Another hypothesis beyond the scope of our study, but undoubtedly worth investigating, is the influence of the genetic characteristics of fox clusters that might lead to a decreased susceptibility to a certain infection. Of note, the genetic background of susceptible populations may influence disease parameters, such as the incubation period or virus shedding, which ultimately may influence the disease dynamics. This is a field, which is receiving increasing attention with studies looking at resistance- and susceptibility-associated alleles [83, 84]. Although empirical data indicate a high variability in terms of resistance and immune response to rabies of raccoon (*Procyon lotor*), such an association is difficult to demonstrate in practice [83–88]. As for the red fox, no data indicate genetic variability linked to resistance to diseases [83–85]. In addition to the factors known from the existing literature, other aspects prevented us from performing this kind of study in our dataset, such as the recent history of rabies and distemper epidemics and the confounding application of oral fox vaccination campaigns. Of note, a speculation based on the association between functional markers and a specific diseases status of individuals collected from the field might have been applicable to the Italian subset alone, due to the absence of positive samples for the two diseases from the other countries involved in this study.

## Conclusions

This study improved our knowledge of the red fox population in the area stretching between the Eastern and Dinaric Alps. Consistently with previous studies, we revealed the lack of a strong genetic structure in the fox population in the study area [31, 33, 34, 72]. Based on our findings we suggest that the disease dynamics in the study area could have been affected by the presence of migrant individuals and of ecological/physical corridors and barriers. Our study also highlights the importance of establishing a coordinated interregional cooperation to identify corridors and barriers with the final shared objective of optimising control efforts. This information could inform the design and implementation of surveillance strategies for infectious diseases in wildlife, better targeting areas deserving increased attention. The study provides an avenue for the interpretation of surveillance data that could be applicable to other wildlife diseases and highlights the need for better understanding the ecology and physiology associated with infections if we want to improve the control of epidemic spread in wildlife populations.

## Supporting information

S1 Fig **Distribution of the genetic groups (A-B-C) in the study area.** Considering structure result K = 3 for the complete dataset, individuals assigned to groups A, B and C are identified with violet, light green and orange dots, respectively. Samples with an assignment probability greater than or equal to 0.7 (Q ≥ 0.7) are shown with dots; admixed individuals are shown with grey squares.
(TIF)

S2 Fig Structure program results. Structure analysis performed on the Italian subset estimated K = 4 as the most likely number of clusters.
(TIF)

S3 Fig Correlogram of the autocorrelation coefficient (r) as a function of distance classes. Correlogram of the whole dataset with distance classes of 10 km. Correlation coefficient (r) is shown in the vertical axis. Error bars bound the 95% confidence interval determined by bootstrap resampling (999 iterations). Upper (U) and lower (L) confidence limits bound the 95% confidence interval for the null hypothesis of no spatial autocorrelation (r = 0) as determined by 999 permutations.
(TIF)

S4 Fig Mantel test. Geographic Distance in km (GGD) on the x-axes and Linear Genetic Distance (LinGD) on the y-axes.
(TIF)

S1 File Material and methods supplemental information. (DOCX)

S1 Dataset Sample table. Table showing in sequence year of collection, country of origin, specific location, disease status, sample ID, list of alleles for each microsatellite locus.
(XLSX)
