# Supplementary figures and images for: Genetic and spatial characterization of the red fox (Vulpes vulpes) population in the area stretching between the Eastern and Dinaric Alps and its relationship with rabies and canine distemper dynamics
Source: PLoS One. 2019 Mar 12;14(3):e0213515. doi: 10.1371/journal.pone.0213515 (PMC6413928; doi:10.1371/journal.pone.0213515)

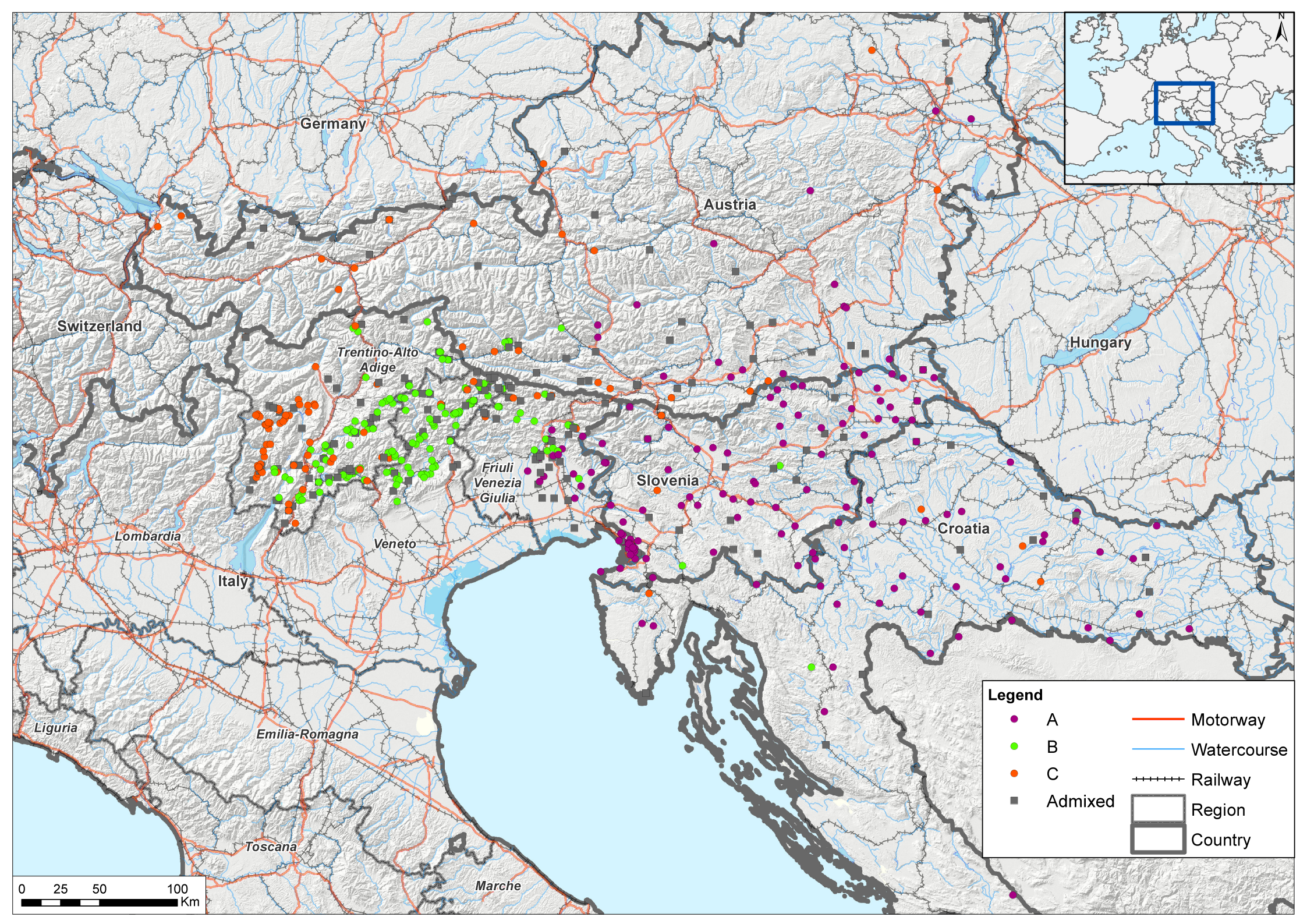

Supplement: S1 Fig — Distribution of the genetic groups (A-B-C) in the study area. Considering structure result K = 3 for the complete dataset, individuals assigned to groups A, B and C are identified with violet, light green and orange dots, respectively. Samples with an assignment probability greater than or equal to 0.7 (Q ≥ 0.7) are shown with dots; admixed individuals are shown with grey squares. (TIF) [file pone.0213515.s001.tif]

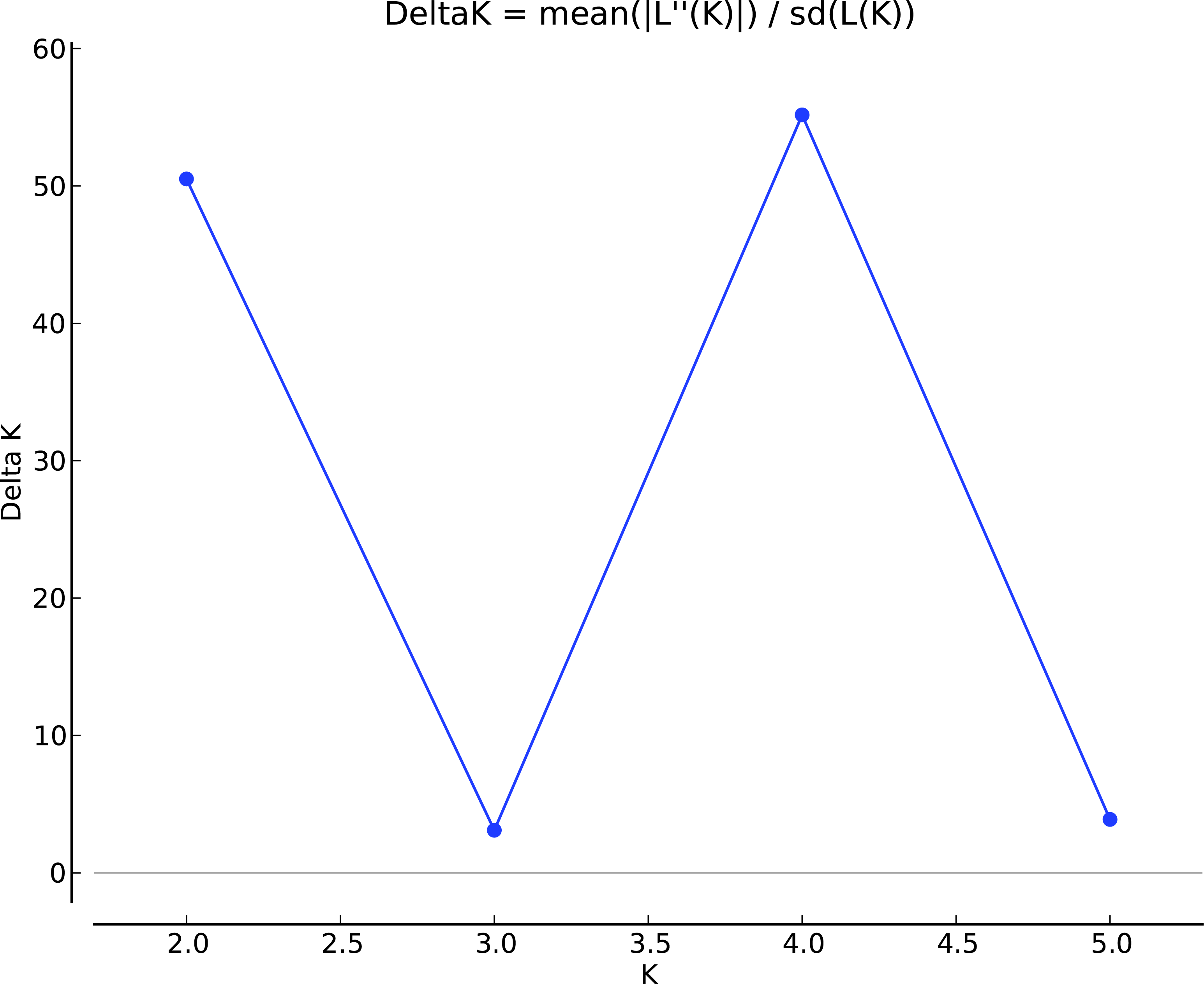

Supplement: S2 Fig — Structure analysis performed on the Italian subset estimated K = 4 as the most likely number of clusters. (TIF) [file pone.0213515.s002.tif]

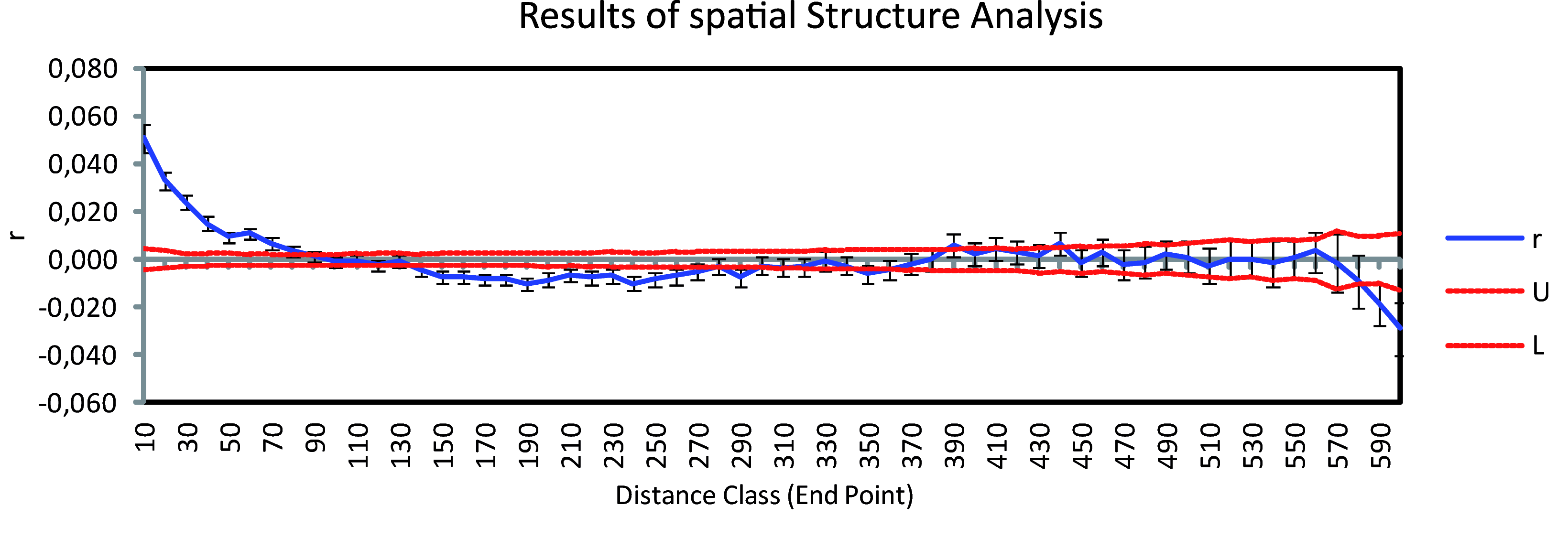

Supplement: S3 Fig — Correlogram of the whole dataset with distance classes of 10 km. Correlation coefficient (r) is shown in the vertical axis. Error bars bound the 95% confidence interval determined by bootstrap resampling (999 iterations). Upper (U) and lower (L) confidence limits bound the 95% confidence interval for the null hypothesis of no spatial autocorrelation (r = 0) as determined by 999 permutations. (TIF) [file pone.0213515.s003.tif]

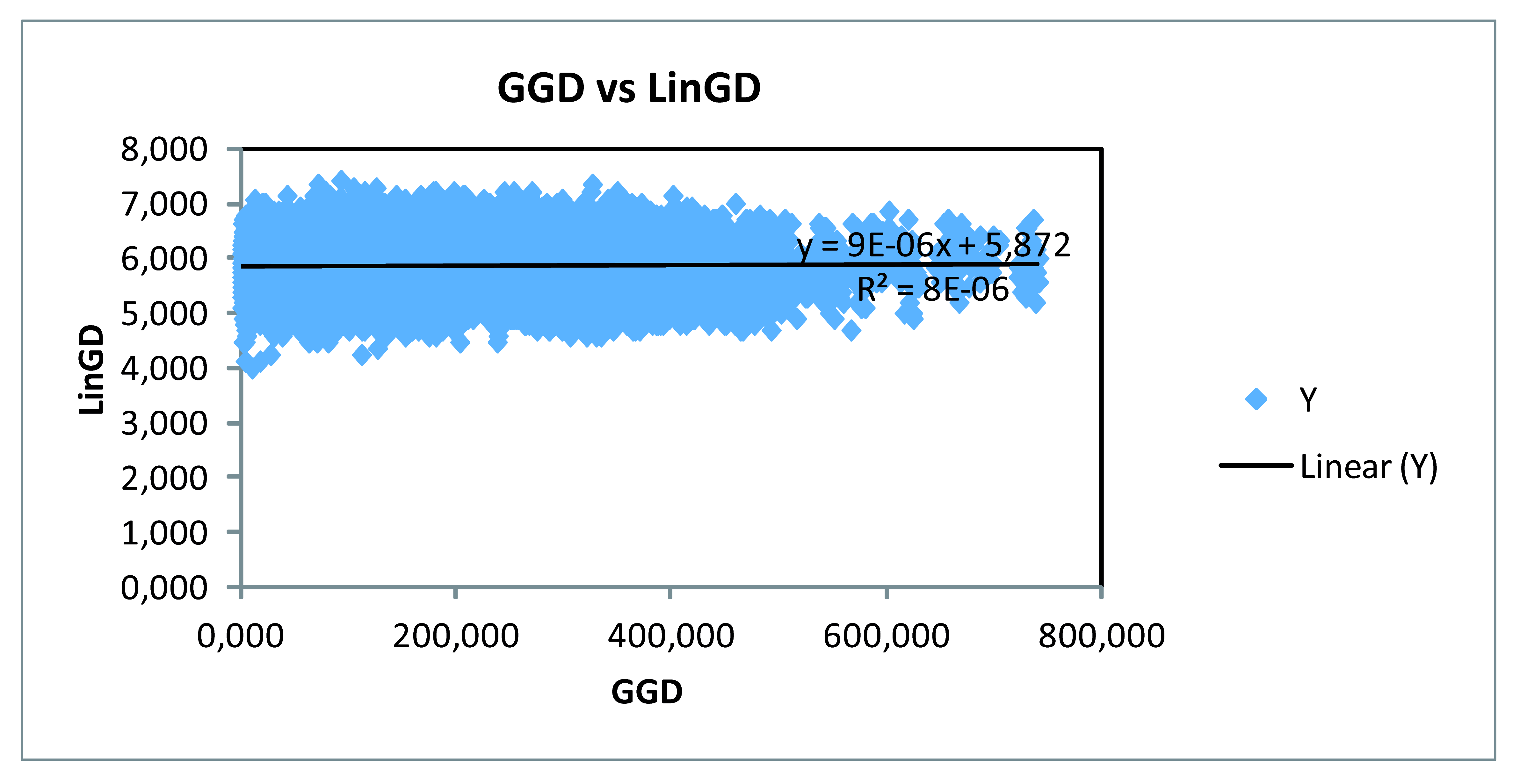

Supplement: S4 Fig — Geographic Distance in km (GGD) on the x-axes and Linear Genetic Distance (LinGD) on the y-axes. (TIF) [file pone.0213515.s004.tif]
