# Supplementary material for: Genetic and spatial characterization of the red fox (Vulpes vulpes) population in the area stretching between the Eastern and Dinaric Alps and its relationship with rabies and canine distemper dynamics
Source: PLoS One. 2019 Mar 12;14(3):e0213515. doi: 10.1371/journal.pone.0213515 (PMC6413928; doi:10.1371/journal.pone.0213515)
Supplement: S1 File — (DOCX) [file pone.0213515.s005.docx]

**Supporting information**

**Study area**

The Italian territory considered includes 5 provinces (Udine, Trieste, Belluno, Bolzano and Trento) of three Italian north-eastern regions (from east to west): Friuli Venezia Giulia, Veneto and Trentino Alto Adige (Fig 1). Elevation ranges from sea level up to approximately 3900 m (Ortles-Cevedale, Trentino Alto Adige), the total area is roughly 39,850 km² and the regions are characterized by very different ecosystems, such as a) forested areas in the Pre-Alpine and Alpine regions (Eastern Alps), b) rural farmlands, c) flatlands with cultivated fields and d) scattered farms and coastlands, both characterized by a more urbanized and industrial environment. This area shares its borders with Slovenia to the east and Austria to the north and is crossed by a number of highways (A22, A23), rivers (i.e. the Tagliamento, the Piave, the Brenta and the Adige rivers), canals and lakes (i.e. Lake Garda).

The Austrian territory covers an area of 83,879 km² , it includes part of the Rhaetian Alps and the whole group of the Eastern Alps (Austrian Alps, Tauern, Carnic and Noric Alps). Three-fifths (3/5) of the country are occupied by the Alpine region; moving eastward, the Eastern Alps descend gradually towards the Carpathians. One third (1/3) of the country located further west consists of a corridor between Germany and Italy, 32-60 km wide. The Alps (Julian Alps, Kamnik-Savinja Alps, Karavanke, Pohorje) also dominate Northern Slovenia along its border with Austria and Italy.

Slovenia covers 20,273 km² and most of the country is hilly or mountainous, with around 90% of the surface located at 200 meters or more above sea level.

Croatia's territory covers 56,594 km2 and the morphology varies from the plains along the Hungarian border to the low mountains and islands. The area of the Black Sea drainage basin covers 62% of the Croatian territory and includes the largest rivers flowing across the country, the Danube, the Sava and the Drava.

**Detection of rabies and canine distemper viruses**

Field samples were screened for rabies infection through the Fluorescent Antibody Test (FAT), as elsewhere described [1]. All positive samples were confirmed in parallel through viral isolation [1] as well as a one-step RT-PCR amplification, as previously described [2].

The detection of canine distemper virus was performed by using the QuantiTect Multiplex RT-PCR kit (Qiagen, Hilden, Germany), following a real-time RT-PCR protocol which amplifies a small region of the N gene coding for the viral nucleoprotein. Primers sequences and protocols are reported by Elia et al., 2006 [3].

**Table A. Sampling frequencies according to Nation.**

| 1. **Italy** | | | | | | | | | | | | | | | | | | | | |
| --- | --- | --- | --- | --- | --- | --- | --- | --- | --- | --- | --- | --- | --- | --- | --- | --- | --- | --- | --- | --- |
|  | Period | |  |  | |  | | Disease status | | |  | | |  | Region | |  | |  | |
|  | 2006-07 | | 2008-09 | 2010-11 | |  | | CDV POS | | Rabies POS  (Italy1+Italy2) | | | NEG |  | Friuli VG* | | Veneto | | Trentino AA** | |
| 379 Samples | 39 | | 106 | 234 | |  | | 148 | | 95(25+70) | | | 136 |  | 96 | | 97 | | 186 | |
| 1. **Austria** | | | | | | | | | | | | | | | | | | | | |
|  | Period | |  |  |  | | Disease status | | | | |  | | |  |  | |  | |  |
|  | 2010 | | 2011 | 2012 |  | | CDV POS | | Rabies POS | | | NEG | | |  |  | |  | |  |
| 98 Samples | - | | 51 | 47 |  | | 8 | | - | | | 90 | | |  |  | |  | |  |
| 1. **Slovenia** | | | | | | | | | | | | | | | | | | | | |
|  | Period | |  |  |  | | Disease status | | | | |  | | |  |  | |  | |  |
|  | 2010 | | 2011 | 2012 |  | | CDV POS | | Rabies POS | | | NEG | | |  |  | |  | |  |
| 86 Samples | 53 | | 33 | - |  | | 10 | | - | | | 76 | | |  |  | |  | |  |
| 1. **Croatia** | | | | | | | | | | | | | | | | | | | | |
|  | Period | |  |  |  | | Disease status | | | | |  | | |  |  | |  | |  |
|  | 2010 | | 2011 | 2012 |  | | CDV POS | | Rabies POS | | | NEG | | |  |  | |  | |  |
| 64 Samples | - | | - | 64 |  | | 7 | | - | | | 57 | | |  |  | |  | |  |
| 627 Samples (Total) | |  |  |  |  | |  | |  | | |  | | |  |  | |  | |  |

*Friuli Venezia Giulia; **Trentino Alto Adige

Sampling frequency according to period, region and disease status for Italy (**A**); sampling frequency according to period and disease status for Austria (**B**), Slovenia (**C**), Croatia (**D**).

**Table B. Genetic differentiation between pairs of red fox clusters (membership probability Q ≥ 0.8).**

|  | Cluster1 | Cluster2 | Cluster3 | Cluster4 |
| --- | --- | --- | --- | --- |
| Cluster1 |  | 0.001 | 0.001 | 0.001 |
| Cluster2 | 0.042 |  | 0.001 | 0.001 |
| Cluster3 | 0.045 | 0.039 |  | 0.001 |
| Cluster4 | 0.042 | 0.039 | 0.047 |  |

Pairwise values of Fst in the lower triangle of the matrix and p-values in the upper triangle, among four clusters composed by individuals with membership probability Q ≥ 0.8 (GenALEx).

**Table C. Alleles and Heterozygosity: complete dataset.**

| **Locus** | **N** | **Na** | **Ne** | **Ho** | **He** | **F** |
| --- | --- | --- | --- | --- | --- | --- |
| **FH2010** | 626 | 4.000 | 2.817 | 0.599 | 0.645 | 0.071 |
| **C04-140** | 625 | 13.000 | 6.025 | 0.810 | 0.834 | 0.029 |
| **C01-424** | 624 | 7.000 | 3.293 | 0.647 | 0.696 | 0.070 |
| **FH2001** | 624 | 23.000 | 5.931 | 0.796 | 0.831 | 0.042 |
| **RF-CPH2** | 625 | 5.000 | 2.143 | 0.509 | 0.533 | 0.046 |
| **FH2328** | 612 | 24.000 | 4.999 | 0.735 | 0.800 | 0.081 |
| **CPH18** | 627 | 14.000 | 4.238 | 0.675 | 0.764 | 0.117 |
| **RF-CXX468** | 627 | 10.000 | 6.226 | 0.796 | 0.834 | 0.045 |
| **FH2848** | 626 | 16.000 | 7.246 | 0.823 | 0.862 | 0.046 |
| **RF-CPH3** | 625 | 16.000 | 9.838 | 0.875 | 0.898 | 0.026 |
| **RF-INU055** | 627 | 10.000 | 4.922 | 0.767 | 0.797 | 0.037 |
| **RF-REN169O18** | 627 | 16.000 | 8.726 | 0.843 | 0.885 | 0.047 |
| **AHT-137** | 627 | 12.000 | 6.872 | 0.813 | 0.854 | 0.048 |
| **RF-REN162C04** | 624 | 12.000 | 7.548 | 0.856 | 0.868 | 0.014 |
| **RF-CXX402** | 626 | 8.000 | 5.034 | 0.738 | 0.801 | 0.079 |
| **AHT-121** | 625 | 16.000 | 7.619 | 0.829 | 0.869 | 0.046 |
| **RF-REN105L03** | 626 | 13.000 | 7.476 | 0.834 | 0.866 | 0.037 |
| **C08-618** | 627 | 11.000 | 5.835 | 0.790 | 0.829 | 0.047 |
| **RF-CPH11** | 626 | 8.000 | 2.236 | 0.548 | 0.553 | 0.009 |
| **RF-CXX-279** | 627 | 13.000 | 4.774 | 0.756 | 0.791 | 0.044 |
| **FH2088** | 626 | 15.000 | 6.368 | 0.762 | 0.843 | 0.096 |

N = No. of Samples; Na = No. of Different Alleles; Ne = No. of Effective Alleles = 1 / (Sum pi^2); Ho = Observed Heterozygosity = No. of Hets / N; He = Expected Heterozygosity = 1 - Sum pi^2; F = (He - Ho) / He

**Table D. Alleles and Heterozygosity: groups A and B (membership probability Q ≥ 0.7).**

| **Group** |  | **N** | **Na** | **Ne** | **Ho** | **He** | **F** |
| --- | --- | --- | --- | --- | --- | --- | --- |
| **Group A** | **Mean** | 262.095 | 11.905 | 5.593 | 0.762 | 0.787 | 0.032 |
|  | **SE** | 0.168 | 0.971 | 0.448 | 0.024 | 0.023 | 0.008 |
|  |  |  |  |  |  |  |  |
| **Group B** | **Mean** | 285.238 | 10.571 | 5.453 | 0.745 | 0.784 | 0.049 |
|  | **SE** | 0.478 | 0.782 | 0.427 | 0.023 | 0.022 | 0.008 |

N = No. of Samples; Na = No. of Different Alleles; Ne = No. of Effective Alleles = 1 / (Sum pi^2); Ho = Observed Heterozygosity = No. of Hets / N; He = Expected Heterozygosity = 1 - Sum pi^2; F = (He - Ho) / He

**Table E. Alleles and Heterozygosity: clusters 1, 2, 3, 4 (membership probability Q ≥ 0.7).**

| **Cluster** |  | **N** | **Na** | **Ne** | **Ho** | **He** | **F** |
| --- | --- | --- | --- | --- | --- | --- | --- |
| **Cluster 1** | **Mean** | 38.952 | 8.286 | 4.904 | 0.732 | 0.756 | 0.035 |
|  | **SE** | 0.048 | 0.598 | 0.428 | 0.030 | 0.025 | 0.016 |
|  |  |  |  |  |  |  |  |
| **Cluster 2** | **Mean** | 57.571 | 8.333 | 4.946 | 0.753 | 0.769 | 0.020 |
|  | **SE** | 0.289 | 0.607 | 0.352 | 0.023 | 0.021 | 0.015 |
|  |  |  |  |  |  |  |  |
| **Cluster 3** | **Mean** | 54.952 | 8.667 | 4.725 | 0.718 | 0.756 | 0.050 |
|  | **SE** | 0.048 | 0.630 | 0.357 | 0.031 | 0.023 | 0.018 |
|  |  |  |  |  |  |  |  |
| **Cluster 4** | **Mean** | 57.952 | 9.190 | 5.437 | 0.750 | 0.779 | 0.035 |
|  | **SE** | 0.048 | 0.639 | 0.423 | 0.027 | 0.026 | 0.017 |

N = No. of Samples; Na = No. of Different Alleles; Ne = No. of Effective Alleles = 1 / (Sum pi^2); Ho = Observed Heterozygosity = No. of Hets / N; He = Expected Heterozygosity = 1 - Sum pi^2; F = (He - Ho) / He

**References**

1. OIE Manual of Diagnostic Tests and Vaccines for Terrestrial Animals 2018. Chapter 2.1.17, Rabies (infection with rabies virus and other lyssaviruses). 2018. Available at: <http://www.oie.int/fileadmin/Home/eng/Health_standards/tahm/2.01.17_RABIES.pdf>.
2. De Benedictis P, De Battisti C, Dacheux L, Marciano S, Ormelli S, Salomoni A, Caenazzo ST, Lepelletier A, Bourhy H, Capua I, Cattoli G. Lyssavirus detection and typing using pyrosequencing. J Clin Microbiol. 2011 May;49(5):1932-8. doi:10.1128/JCM.02015-10.
3. Elia G, Decaro N, Martella V, Cirone F, Lucente MS, Lorusso E, Di Trani L, Buonavoglia C. Detection of canine distemper virus in dogs by real-time RT-PCR. J Virol Methods. 2006 Sep;136(1-2):171-6.
